# Monolith affinity chromatography for the rapid quantification of a single‐chain variable fragment immunotoxin

**DOI:** 10.1002/jssc.201800257

**Published:** 2018-07-01

**Authors:** Peter Satzer, Ralf Sommer, Johanna Paulsson, Agnes Rodler, Romana Zehetner, Klaus Hofstädter, Christoph Klade, Alois Jungbauer

**Affiliations:** ^1^ Department of Biotechnology University of Natural Resources and Life Sciences Vienna Austria; ^2^ Department of Applied Biochemistry Lund University Lund Sweden; ^3^ AOP Orphan Pharmaceuticals AG Vienna Austria; ^4^ Austrian Centre of Industrial Biotechnology Vienna Austria

**Keywords:** affinity chromatography, antibody‐drug conjugates, immunotoxins, monoliths, process analytical tools

## Abstract

We developed a novel analytical method for concentration determination of tandem single‐chain antibody diphtheria toxin (immunotoxin). The method is based on polymethacrylate monoliths with Protein L ligands as the binding moiety. Different buffers were tested for elution of the Protein L‐bound immunotoxin and 4.5 M guanidinium hydrochloride performed best. We optimized the elution conditions and the method sequence resulting in a fast and robust method with a runtime <10 min. Fast determination of immunotoxin is critical if any process decisions rely on this data. We determined method performance and a lower limit of detection of 27 μg/mL and a lower limit of quantification of 90 μg/mL was achieved. The validity of the method in terms of residual analysis, precision, and repeatability was proven in a range from 100 to 375 μg/mL. The short runtime and ease of use of a high‐performance liquid chromatography method is especially useful for a process analytical tool approach. Bioprocesses related to immunotoxin where fermentation or other process parameters can be adjusted in accordance to the immunotoxin levels will be benefited from this method to achieve the highest possible purity and productivity.

**Article Related Abbreviations**
DT: diphtheria toxin
HPMAC: high‐performance monolith affinity chromatography
scFv: single‐chain antibody

## 1 INTRODUCTION

Monoliths offer a way for the rapid determination of biomolecules such as immunotoxins. In this case a fast and robust method was developed for in‐process control for an immunotoxin. A bivalent immunotoxin consisting of two tandem single‐chain antibodies (scFv) and a truncated diphtheria toxin (DT) intended for the treatment of CD3‐positive peripheral T‐cell lymphoma (leukemic, nodal, and extranodal) and the treatment of cutaneous T‐cell lymphoma 1, 2. The immunotoxin is expressed in *Pichia pastoris* 3, 4, 5, 6. For rapid in‐process control a fast method with high separation efficiency and high selectivity is required to be able to determine the compound in the culture supernatant. The construct contains a mutated DT, preceded by an alanine residue (A) and truncated at amino acid residue 390. The mutations of the diphtheria removed both glycosylation sites within the toxin that would reduce bioactivity when expressed in *P. pastoris*. The antibody part consists of two tandem scFv antibody fragments from the antihuman anti‐T cell antibody UCHT1 directed at the CD3ε epitope that is only expressed on T cells 7, 8, 9, 10, 11. For clinical and preclinical studies 12, 13 the up‐ and downstream processing of A‐dmDT390‐bisFv(UCHT1) was optimized in previous study 5, 14. Sole bottleneck in the GMP production and process development of A‐dmDT390‐bisFv(UCHT1) is the time‐consuming in‐process control during up‐ and downstream processing, which consists of a preparative fractionation by size exclusion and analysis of the fractions by SDS‐PAGE. The Fv(UCHT1) binds to protein L enabling affinity chromatography of the construct, avoiding time‐consuming methods, labeling, or toxin‐specific ligands 15, 16, 17. Monoliths have been proven to work reliably and fast in complex samples for antibody, toxins, antibody conjugates or even larger molecules 18, 19, 20, 21, 22, 23, 24 and affinity chromatography is widely established for antibodies and antibody conjugates 20, 25, 26, 27, 28, 29, 30, 31, 32, 33. Using protein L‐functionalized monoliths 20 a new method was developed for the measurement of the immunotoxin by changing elution parameters. After method development, we determined method performance and stability according to common procedures 19, 34. The newly developed method was able to reduce the measurement time from half a day to approximately 30 min including sample preparation and is, therefore, suitable for an in‐process control for in‐line monitoring.

## 2 MATERIALS AND METHODS

All chemicals used for buffer preparation were purchased from Merck, Darmstadt, Germany.

### 2.1 scFv immunotoxin fusion protein

Two different samples containing scFv immunotoxin fusion protein were available for experiments, one purified standard with approximately 400 μg/mL protein and one sample from end of the fermentation containing target protein and host‐cell impurities. These were kindly provided by AOP Orphan Pharmaceuticals Vienna, Austria.

### 2.2 Electrophoresis

For electrophoresis, NuPage®‐Bis‐Tris 4–12% gradient gels (Invitrogen, Carlsbad, CA, USA) in the XCell II™ Mini‐Cell (Invitrogen) were used according to the manufacturer. Samples were prepared with NuPage®‐LDS‐sample buffer supplemented with 0.1 M dithiothreitol before loading onto the gel. SDS‐PAGE was performed with MES SDS running buffer, prepared as described by the supplier, at 200 V and 400 mA for 50 min. The gel was stained with silver solution (6 mM silver nitrate, 7 mM formaldehyde) for 20 min. The gel was washed with developing solution (236 mM sodium carbonate, 4 mM formaldehyde) for 5–10 min by changing the solution every 2–3 min. Prior scanning gel was put for 10 min in stop solution (39 mM EDTA).

### 2.3 Western blot

An XCell II™ Blot system (Life Technologies), blotting filter paper (BioRad), protran nitrocellulose transfer membrane with the pore size of 0.2 μm (Schleicher and Schuell purchased at GE Healthcare), and transfer buffer (50 mM sodium tetraborate decahydrate, 0.1% w/v SDS, 20% methanol) were used. Transfer was done at 40 V, 200 mA/gel for 2 h. The membrane was washed with PBS buffer (140 mM sodium chloride, 3 mM potassium chloride, 10 mM disodium phosphate, and 2 mM dihydrate monopotassium) supplemented with 0.1% w/v Tween 20. Washing and incubation were done for 15 min during which the washing buffer was changed twice. After blotting, the membrane was washed and then incubated with blocking buffer (PBS buffer with 3% w/v skim milk powder) overnight at 4°C. The membrane was incubated for 2 h with PBS buffer with 1% w/v of skim milk powder and IgG DT polyclonal antibody from a goat (diluted 1:1000) for labeling. The secondary antibody was applied during a wash with PBS buffer supplemented with 1% w/v of skimmed milk powder and anti‐goat IgG‐alkaline phosphatase antibody produced in a rabbit (diluted 1:10000). After washing, a Lumi‐Phos‐Reagent (Pierce Biotechnology) was used to visualize the result in a LumiImager Workstation (Roche Molecular Biochemical).

### 2.4 Chromatography

All chromatographic experiments were performed on an Agilent Series 1100 System (Agilent, Waldbronn, Germany) consisting of a 96‐well plate automatic liquid sampler (WP ALS) for injection, a degasser, a quaternary pump, and a diode array detector. All buffers were filtered with a 0.1 μm nitrocellulose filter (Millipore). The HPLC system was used at room temperature. The autosampler was used with a built‐in sample loop of 100 μL. An additional loop was installed to inject a maximum volume of 900 μL. The ChemStation for LC 3D sys (Rev. B. 04.03) software was used for data acquisition and control.

### 2.5 SEC

A TSKgel G3000SWXL column with a TSKgel SWXL guard column (Tosoh Bioscience, Stuttgart, Germany) was used. Phosphate buffer (150 mM potassium‐phosphate buffer with pH 6.5 containing KH_2_PO_4_ and K_2_HPO_4_) was used as running buffer. The SEC was performed with a flow rate of 0.4 mL/min over 65 min. Chromatograms were recorded at 210 nm wavelength.

### 2.6 High‐performance monolith affinity chromatography (HPMAC)

For capturing of immunotoxins a CIM® r‐Protein L disk (BIA Separations, Ljubljana, Slovenia) was used. Equilibration was performed with a sodium phosphate buffer (1 M NaCl, 30 mM sodium phosphate, pH 7.5, containing Na_2_HPO_4_ and NaH_2_PO_4_). Target protein was eluted with elution buffer containing 4.5 M GuHCl (Guanidine Hydrochloride LAB 104220). The affinity chromatography was performed with a flow rate of 1.0 mL/min. Chromatograms were measured at 280 nm wavelength. HPMAC method starts with sample injection followed by equilibration with running buffer for the separation of nonbinding proteins. Afterwards bound proteins were removed by flushing with elution buffer (4.5 M GuHCl). Finally an equilibration with equilibration buffer was performed.

#### 2.6.1 Method development

To determine appropriate elution volumes different wash times were tested after injection. The elution was done with 4.5 M GuHCl with varying elution volumes between 0.3 and 2.5 mL. All experiments for method development were performed at a flow rate of 1 mL/min. After the elution, the column was then regenerated with 6.0 M GuHCl and re‐equilibrated for the next sample with equilibration buffer for 2.5 min.

#### 2.6.2 Calibration/validation of CIM® r‐Protein L disk with guanidine hydrochloride LAB

The calibration and validation were done with a standard diluted with deionized water to 16 different concentrations ranging between 5 and 400 μg/mL (5, 25, 50, 75, 100, 125, 150, 175, 200, 225, 250, 275, 300, 325, 350, 375, and 400 μg/mL). In total seven independent dilution series were made of which three replicates, three intraday calibrations, and one that was made to get measurements from three different days and monoliths. This setup was done according to guidelines from the International Conference on Harmonization, ICH Q2 (R1) (5) 35. The sequence in which each sample was run in each calibration was randomized. Adsorption was monitored at 280 nm, where the flow‐through was calculated from the chromatogram between 0.1 and 1.4 min and for bound proteins between 1.46 and 5 min. The protein concentration of the standard was determined by the measurement of absorbance at 276 nm and was calculated with the mass extinction coefficient of 1.63 (276 nm).

#### 2.6.3 Formation of immunotoxin aggregates

Immunotoxin aggregates were formed by heating of immunotoxin standard solutions and by adding GuHCl to the standard solution. The heat‐formed aggregates were created by heating of the standard solution to 55°C for either 35 min or 10 min in a water bath. For aggregates induced by the addition of GuHCl, 6 M of GuHCl was added three parts to one part resulting in a four times dilution.

## 3 RESULTS AND DISCUSSION

### 3.1 Method development

**Figure 1:**
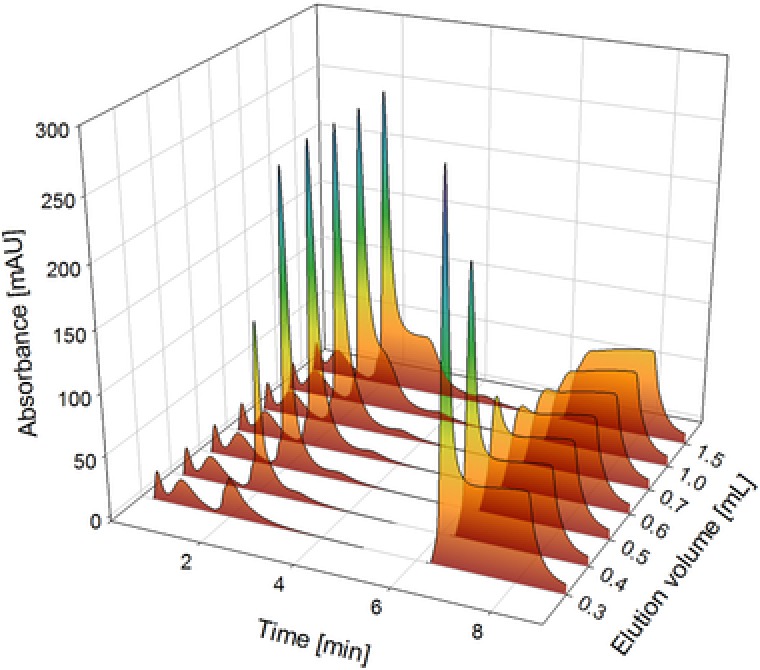
100 μL standard injected on CIM® r‐Protein L disk, first elution with different volumes of 4.5 M GuHCl and regeneration with 2 mL of 6 M GuHCl. Elution starts at 1.8 min, regeneration at 6.2 min

For the fast determination of the immunotoxin a novel affinity chromatographic method based on the CIM® r‐Protein L disk was developed. For initial development, we kept flow rate (1 mL/min) and injection volume (100 μL) constant while the elution conditions were optimized. We tested HCl (at pH 2 and 3) without salt as well as with the addition of either 0.1 M glycine, 2 M NaCl or 4 M ammonium sulfate. Additionally an elution buffer with 1 to 6 M GuHCl was tested. Only GuHCl (4.5 M) was an effective elution buffer which was then tested for different elution volumes between 0.3 and 1.5 mL (Figure 1), the elution peak being at min 2, and a regeneration with 6.0 M GuHCl at min 6.5. The elution peak does not significantly change with higher elution volumes than 1.0 mL and the regeneration shows no signal for 1.0 mL elution volume either, so this is what we used for further experiments.

We determined the method performance by calculating the confidence interval of the calibration curves as well as a test for linearity and lack‐of‐fit test. We defined the concentration range to be valid if the data indicated no significant difference on a 95% level for linearity, a 99% level for lack‐of‐fit, and a simultaneous 95% confidence interval. Also, the lowest concentration had to be above the LOD and LOQ. Calculation of these values were based on the SD of the response and the slope according to recommendations by ICH Q2 (R1) 35, 36, see Equations (1) and (2), where DL is the lower LOD and QL is the lower LOQ.

(1) $$\mathrm{DL}=\frac{3.3\sigma }{k},$$

(2) $$\mathrm{QL}=\frac{10\sigma }{k},$$

where *k* is the slope of the calibration curve and *σ* the SD of the response. The SD of the response was determined by using data of the residuals of the calibration curve 37. We made three dilution series with concentrations between 5 and 400 μg/mL for intraday variation, one dilution series measured three times on consecutive days for sample stability, and three dilution series on three consecutive days for interday variation. After each trial, the valid concentration range was narrowed down because of the restrictions we set for linearity, lack of fit, and confidence interval. Only the valid concentrations were prepared for the next set of experiments. Performance parameters of valid range, LOD, LOQ, linearity, and corresponding fit are presented in Table 1. After the narrow concentration range was determined, all data from that range from all seven dilutions measured were combined to calculate the final valid calibration.

**Table 1:** Method performance in terms of intraday, interday, and sample stability. The valid calibration range is calculated from all the data

Curves	Range (μg/mL)	LOD (μg/mL)	LOQ (μg/mL)	*R* ^2^	Regression equation
Intraday	100–400	28.89	96.31	0.990	*y* = 8.09 x – 324.5
Stability	100–375	13.26	44.20	0.999	*y* = 8.46 x – 345.4
Interday	100–375	18.40	61.35	0.996	*y* = 7.93 x – 305.9
Valid calibration	100–375	27.04	90.14	0.988	*y* = 8.05 x – 314.9

**Figure 2:**
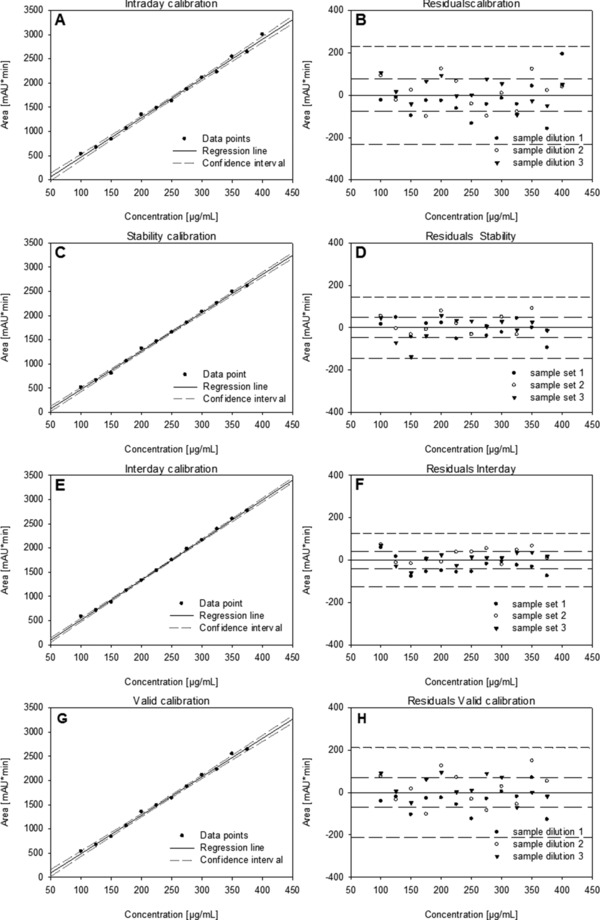
Panel A, C, E, and G show the calibration and confidence interval of intraday, stability, interday, and valid concentration range, each data point was measured in triplicate. Panel B, D, F, and H show the corresponding residuals

The experimental data including the fitted curves are plotted in Figure 2 and divided in calibration curves (Figure 2A, C, E, and G) and residuals (Figure 2B, D, F, and H). The initial concentration range tested for this experiment was 5–400 μg/mL, but concentrations had to be excluded from the valid range because of the defined criteria of LOQ, LOD, linearity, and confidence interval and the figures only show the valid concentration range for comparability. The performance parameters of the methods are a valid concentration range of 100–375 μg/mL with a LOD of 29 μg/mL and a runtime of 10 min. The upper LOQ of 375 μg/mL might be because of saturation effects on the analytical column and dilution of samples with higher concentrations is therefore necessary. In comparison to the traditional method of SEC fractionation followed by SDS‐gel electrophoresis, this is a significant improvement, saving in cost, and making measurement more convenient and robust by transferring it to HPLC.

### 3.2 Comparison of monoliths

**Figure 3:**
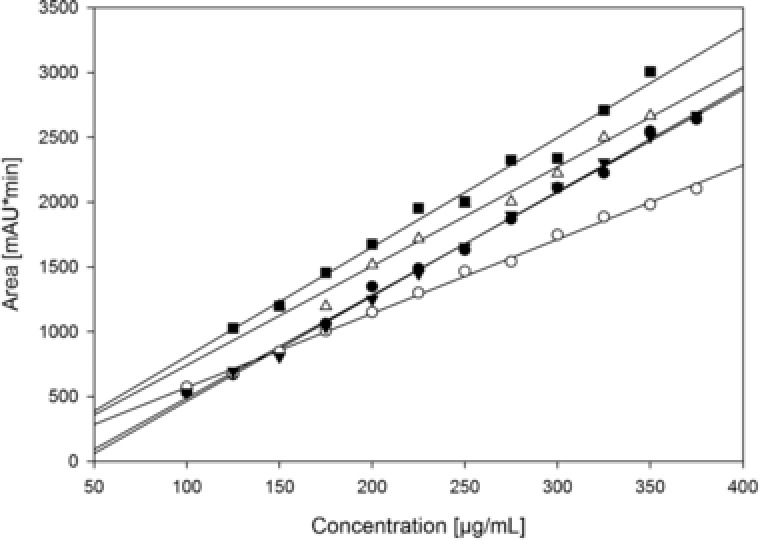
Analysis and calibration curves of immunotoxin on CIM® r‐Protein L disks. Data points consist of three independent replicas

To ensure stability of the method from one monolith to the next, we compared calibration curves from 100 to 375 μg/mL for five different monoliths using the same method as described before (Figure 3). The data suggest a high variation from one monolith to the next, which can also be seen if we recalculate the valid concentration range for each monolith individually as well as LOQ and LOD (Table 2). Some monoliths show similar performance (monolith 1 and 3) but for others, this is not the case. While monoliths 1, 3, 4, and 5 show roughly the same slope, monolith 2 shows a shallower curve. For this monolith we also detected flow‐through during loading, explaining a shallower curve. This might be because of channeling in the monolith due to a bypass canal 38. For the use of this method we suggest to perform a calibration whenever the column is changed to ensure consistent data collection. Additionally, the slope of the calibration can be used as quality criteria for the monolith, indicating maybe bypass canals in the monolith or other product flaws.

**Table 2:** Range, LOD, LOQ, *R* ^2^, and regression equation of all monolith calibrations are shown in Figure 3

Curves	Range (μg/mL)	LOD (μg/mL)	LOQ (μg/mL)	*R* ^2^	Regression equation
Monolith 1	100–375	27.04	90.14	0.988	*y* = 8.05 *x*−314.9
Monolith 2	100–375	21.97	73.97	0.993	*y* = 5.50 *x*−35.2
Monolith 3	100–375	19.15	63.82	0.995	*y* = 8.08 *x*−338.4
Monolith 4	150–350	43.17	130.83	0.982	*y* = 7.66 *x*−25.9
Monolith 5	125–350	39.57	119.92	0.987	*y* = 8.44 *x*−35.6

#### 3.2.1 Selectivity – Determination of Immunotoxin in fermentation supernatant

**Figure 4:**
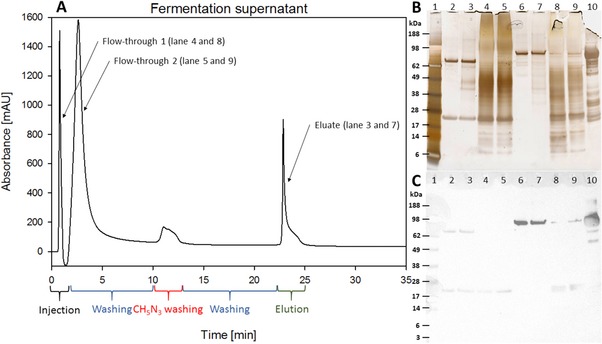
Panel A: Analysis of immunotoxin after the end of fermentation on CIM® r‐Protein L disk with 500 μL sample volume. The collected fractions were analyzed by SDS‐PAGE. The elution time is from 23 to 25 min, the peak of the target protein is marked with an arrow (Eluate). Panel B: SDS‐PAGE lanes are: lane 1: molecular weight standard, lane 2 and 3: reference and eluate (reduced), lane 4 and 5 flow through (reduced), lane 6 and 7: reference and eluate (non‐reduced), lane 8 and 9: flow through (reduced). Lane 10 shows the unreduced starting material. Panel C shows the corresponding western blot with an anti‐DT antibody

To use HPMAC as analytical at‐line method for immunotoxin determination during the whole process of upstream and downstream some modifications were required. Product concentrations at the start of fermentation were lower than the LOQ and we tried to compensate for that by the use of larger sample volumes (500 μL) and therefore longer runtimes. Figure 4A shows an affinity chromatography of the immunotoxin at end of fermentation with increased sample volume.

Flow‐through of unbound proteins was tailing significantly, so to get complete and faster separation of flow‐through and elution peak an extra washing step was performed after 10 min retention time (2 M GuHCl buffer). Product identity was confirmed by SDS‐PAGE and purity was comparable to the reference standard (Figure 4B). Bands at 25 and 75 kDa in the reference and eluate correspond to the size of the reduced immunotoxin, bands at 96 kDa correspond to the unreduced monomer. Additional bands visible in the reference standard that are not visible in the sample are most likely because of a higher concentration of the reference standard. The method showed good performance in separating the immunotoxin from other components, enabling the measurement of fermentation samples. Using larger volumes enables the method to be useful from mid fermentation through end of fermentation and in the downstream processing as in‐process control, where expected concentrations range from 40 to 150 μg/mL in fermentation and higher concentrations for the downstream processing 5, 6, 14. In comparison to the standard method of size‐exclusion fractionation coupled with SDS‐gel electrophoresis, our method drastically improves the necessary time for the analysis and also reduces complexity. We maintained high selectivity and achieved LOQ and LODs that are compatible with the needs of a real‐world immunotoxin process for fast and easy at‐line quantification.

### 3.3 Sensitivity to aggregates

**Figure 5:**
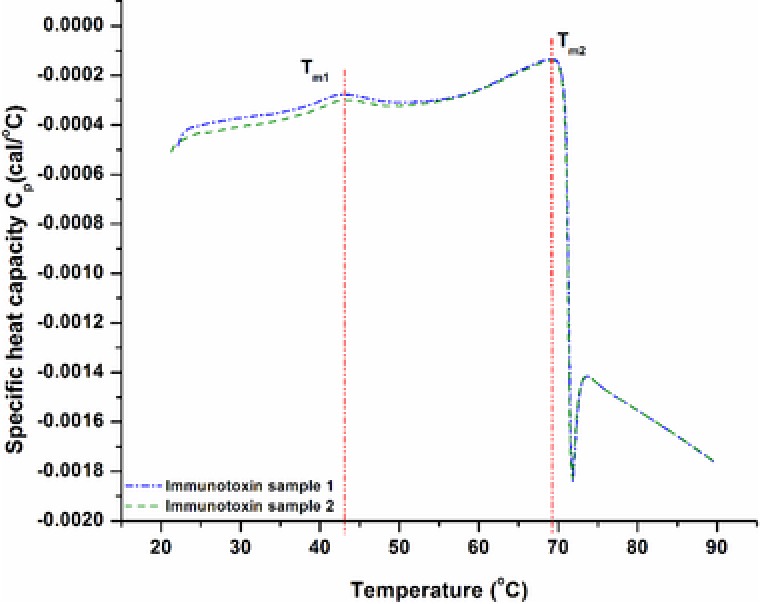
Two replica measurements of immunotoxin by differential scanning calorimetry. T_m1_ and T_m2_ indicated the melting temperatures of the immunotoxin

After selectivity was proven, we wanted to test how the method reacts to immunotoxin‐aggregates. Specifically, if the method could selectively detect the immunotoxin monomer, or if aggregates are always picked up by the method as well. To prepare an aggregate standard, we tried aggregate induction of the reference by heat or by GuHCl treatment. GuHCl led to complete denaturation but no aggregate formation occurred, so we determined the melting temperature of the immunotoxin by differential scanning calorimetry and found two melting points (at 43 and 70°C, Figure 5). For temperatures over 70°C we saw complete denaturation of the immunotoxin. We therefore used temperatures between 43 and 70°C to induce partially unfolded proteins to aggregate with the aim to lose as little natural structure as possible.

**Figure 6:**
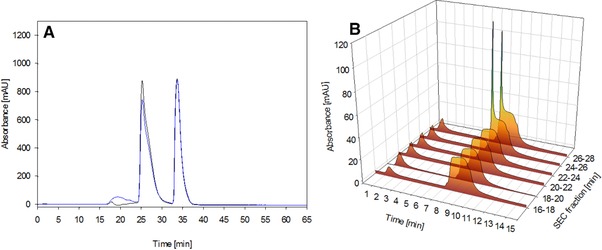
Panel A: SEC with 100 μL reference standard (black), aggregated standard (blue), panel B shows the results of analytical CIM® r‐Protein L disk chromatography for fractions of the SEC chromatogram

Treatment of the reference by heat at 55°C produced higher aggregate levels (Figure 6) without compromising the complete structure of the immunotoxin while lowering the concentration of immunotoxin monomer. To see if the produced aggregates can in fact bind to the column or not, we fractionated the aggregates and the monomer and loaded them on the CIM® r‐Protein L disk. We observed no elution signal for fractions containing aggregates, but clear elution signals for fractions containing monomers. While heat‐induced aggregates might lead to structurally similar but nonidentical aggregates, we believe that we mitigated that risk by selecting an aggregation method preserving the natural conformation as best as we could while still inducing aggregate formation. We conclude that aggregates formed in this study are not able to bind to the CIM‐disks and therefore will not be detected by our method.

## 4 CONCLUDING REMARKS

A fast in‐process control method for the determination of an immunotoxin was developed. This method is based on affinity chromatography with protein L immobilized on the CIM® disk. Determination of performance of the method showed detection and quantification limits of 27 and 90 μg/mL as well as the required robustness, precision, and repeatability within a concentration range of 100–375 μg/mL. The method has a runtime of 10 min per sample for high‐purity samples, and about 30 min for low‐purity samples, and is therefore usable for at‐line measurements for process control, especially in comparison to current technology where analytics take about a day. We demonstrated the selectivity of the method using real‐live fermentation samples and SDS‐PAGE analysis. The method is selective for intact monomers, allowing real‐time quantification of the desired final monomer product. Also, this method can be envisioned to be upscaled as a purification step eliminating aggregates by a bind‐elute process step. As an analytical method it can follow the production of immunotoxins from fermentation through downstream and is therefore suitable for an at‐line measurement guiding a process analytical tool‐approach for production. As a preparative method, it could be combined with an affinity chromatography with a CD3ε. Combination of CD3ε receptor and our method based on protein L only detects proteins with intact scFv and intact DT region.

## CONFLICT OF INTEREST

The authors have declared no conflict of interest.
